# Epidemic Years Increase Hospitalization but Not Mortality Among Laboratory-Confirmed Dengue Cases in Mexico: A Nationwide Surveillance Study, 2020–2025

**DOI:** 10.3390/microorganisms14091920

**Published:** 2026-08-31

**Authors:** Leticia Jaimes Betancourt, Alfonso Vallejos Parás, Porfirio Felipe Hernández Bautista, David Alejandro Cabrera Gaytán, Bernardo Cacho Díaz, Adriana Josefina Toríz Saldaña, Lumumba Arriaga Nieto, Óscar Cruz Orozco, Gabriel Valle Alvarado, Mónica Grisel Rivera Mahey, Mónica Alethia Cureño Díaz, Víctor Gómez Bocanegra, Héctor Alonso Téllez Medina

**Affiliations:** 1 Unidad de Medicina Familiar No. 7, Instituto Mexicano del Seguro Social, Mexico City 14370, Mexico; leticia.jaimesb@imss.gob.mx; 2 Coordinación de Vigilancia Epidemiológica, Instituto Mexicano del Seguro Social, Mexico City 03100, Mexico; david.cabrerag@imss.gob.mx (D.A.C.G.); adriana.toriz@imss.gob.mx (A.J.T.S.); lumumba.arriaga@imss.gob.mx (L.A.N.); oscar.cruzo@imss.gob.mx (Ó.C.O.); gabriel.valle@imss.gob.mx (G.V.A.); monica.riverama@imss.gob.mx (M.G.R.M.); 3 Coordinación de Calidad de Insumos y Laboratorios Especializados, Instituto Mexicano del Seguro Social, Mexico City 07760, Mexico; 4 Instituto Nacional de Cancerología, Secretaría de Salud, Mexico City 14080, Mexico; bernardocacho@doctor.com (B.C.D.); mcurenod@incan.edu.mx (M.A.C.D.); 5 Facultad de Medicina, Universidad Nacional Autónoma de México, Mexico City 04510, Mexico; vgomez@facmed.unam.mx (V.G.B.); hector.tellez@facmed.unam.mx (H.A.T.M.)

**Keywords:** dengue, arboviruses, hospitalization, mortality, epidemics, chronic kidney disease, diabetes mellitus, Mexico, epidemiological surveillance

## Abstract

Dengue is a major public health problem in Mexico, where large epidemics have occurred during recent years. Although demographic and clinical predictors of severe dengue have been widely studied, the impact of epidemic transmission periods on hospitalization and mortality remains poorly understood. This study evaluated the association between epidemic years and hospitalization and mortality among laboratory-confirmed dengue cases in Mexico. A nationwide retrospective analytical study was conducted using laboratory-confirmed dengue cases reported through the Mexican National Epidemiological Surveillance System between 2020 and 2025. Years were classified as epidemic (2023–2024) or non-epidemic (2020–2022 and 2025) according to official endemic channel analyses. Hospitalization and mortality were evaluated as primary outcomes. Multivariable logistic regression models were developed to identify factors independently associated with hospitalization and mortality. A total of 235,226 laboratory-confirmed dengue cases were included, of whom 90,661 (38.5%) were hospitalized and 1604 (0.68%) died. Overall, 170,819 cases (72.6%) occurred during epidemic years. Hospitalization was more frequent during epidemic years than during non-epidemic years (40.2% vs. 34.1%; crude OR 1.30, 95% CI 1.27–1.32; *p* < 0.001), whereas mortality did not differ significantly (0.68% vs. 0.69%; crude OR 0.99, 95% CI 0.89–1.11; *p* = 0.919). In multivariable analysis, epidemic-year status remained independently associated with hospitalization (aOR 1.26, 95% CI 1.24–1.29; *p* < 0.001) but not with mortality (aOR 1.00, 95% CI 0.90–1.12; *p* = 0.946). Chronic kidney disease showed the strongest independent association with both hospitalization (aOR 5.36, 95% CI 4.33–6.65) and mortality (aOR 6.98, 95% CI 5.23–9.32). Advanced age and other underlying comorbidities were also independently associated with adverse outcomes. Epidemic years were associated with increased hospitalization among laboratory-confirmed dengue cases in Mexico but were not associated with increased mortality. Mortality was more strongly associated with age and underlying comorbidities, particularly chronic kidney disease. These findings highlight the importance of strengthening hospital preparedness during epidemic periods while prioritizing clinical monitoring of high-risk patients.

## 1. Introduction

Dengue is the most important mosquito-borne viral disease affecting humans and represents a major public health challenge in tropical and subtropical regions worldwide. The disease is caused by four antigenically distinct dengue virus serotypes (DENV-1 to DENV-4) and is transmitted primarily by *Aedes aegypti* mosquitoes. Clinical manifestations range from asymptomatic infection and mild febrile illness to severe disease characterized by plasma leakage, hemorrhage, organ dysfunction, and death. During the last decades, dengue incidence has increased substantially due to urbanization, population growth, globalization, climate variability, and the geographic expansion of mosquito vectors, resulting in recurrent epidemics and an increasing burden on healthcare systems worldwide [1,2].

The Region of the Americas has experienced an unprecedented increase in dengue transmission in recent years, with several countries reporting record numbers of cases and outbreaks. Mexico is among the countries with the highest dengue burden in the region and has experienced substantial fluctuations in transmission intensity over time [3]. Between 2023 and 2024, Mexico experienced major dengue epidemics characterized by a marked increase in reported cases and substantial changes in circulating DENV serotypes, including the increasing predominance of DENV-3 [3,4,5], which became the predominant serotype in several regions during a period characterized by a marked increase in laboratory-confirmed cases. These outbreaks generated significant pressure on healthcare services and highlighted the need to better understand the determinants of severe clinical outcomes during periods of intense transmission.

Several demographic and clinical factors have been associated with hospitalization and mortality among dengue patients. Previous studies conducted in Mexico and other endemic countries have identified advanced age, diabetes mellitus, chronic kidney disease, hypertension, and hemorrhagic manifestations as important predictors of severe disease and death [6,7,8,9]. In particular, Fonseca-Portilla et al. reported that comorbidities and clinical manifestations were strongly associated with hospitalization and mortality among dengue patients in Mexico [6]. More recently, national analyses have confirmed the relevance of chronic non-communicable diseases and underlying medical conditions in determining adverse outcomes among laboratory-confirmed dengue cases [7,8,9].

Although individual-level risk factors for severe dengue have been extensively studied, less attention has been paid to the potential effect of epidemic intensity on clinical outcomes. Periods of increased transmission may influence healthcare-seeking behavior, increase demand for hospital services, modify admission practices, and potentially affect patient outcomes. However, it remains unclear whether dengue patients diagnosed during epidemic years have a greater risk of hospitalization or death compared with those diagnosed during non-epidemic periods. Understanding this relationship is essential for improving preparedness strategies, optimizing resource allocation, and strengthening the response capacity of healthcare systems during dengue epidemics.

Therefore, the aim of this study was to evaluate the association between epidemic years and the risk of hospitalization and mortality among laboratory-confirmed dengue cases reported in Mexico between 2020 and 2025. Additionally, we assessed the contribution of demographic characteristics, comorbidities, virological factors, and healthcare coverage status to adverse clinical outcomes in a nationwide population of laboratory-confirmed dengue cases.

## 2. Materials and Methods

### 2.1. Study Design and Data Source

A retrospective cross-sectional analytical study was conducted using secondary data obtained from the historical open access databases of vector-borne diseases maintained by the Mexican National Epidemiological Surveillance System and publicly available through the Mexican Ministry of Health [10]. These databases contain anonymized records of laboratory-confirmed dengue cases reported nationwide.

### 2.2. Study Population

The study included all laboratory-confirmed dengue cases reported in Mexico between 2020 and 2025. For each study year, the most recent publicly available surveillance database cut-off was used: 31 December 2020; 30 December 2021; 29 December 2022; 27 December 2023; 4 December 2024; and 26 December 2025. Therefore, the surveillance datasets did not necessarily contain complete records through 31 December for all study years.

Dengue is a nationally notifiable disease in Mexico, and all suspected cases meeting the operational case definition established by the Ministry of Health must be reported through the National Epidemiological Surveillance System (SINAVE) [11].

For the present study, only cases with laboratory confirmation were included. Eligible records were required to have a reported laboratory sample and a definitive laboratory result confirming dengue infection according to national surveillance guidelines. Laboratory confirmation was established through reverse transcription polymerase chain reaction (RT-PCR), NS1 antigen detection, viral isolation, or serological testing, depending on the diagnostic algorithms in force during the study period. According to national dengue surveillance procedures, laboratory testing is performed in 30% of notified dengue cases without warning signs, whereas 100% of cases with warning signs or severe dengue are targeted for laboratory testing [11]. This sampling strategy remained unchanged throughout the 2020–2025 study period. Consequently, the present analysis includes laboratory-confirmed cases and does not represent a random sample of all notified dengue cases.

Because all eligible laboratory-confirmed dengue cases available in the national surveillance database were included, a census of records was analyzed rather than a sample. No additional exclusion criteria were applied.

### 2.3. Variables

The primary outcomes were hospitalization and mortality. Hospitalization was defined as admission to a healthcare facility due to dengue infection, whereas mortality was defined as death occurring among laboratory-confirmed dengue cases registered in the surveillance system.

Independent variables included sex, age, age group (<5, 5–14, 15–24, 25–44, 45–64, and ≥65 years), diabetes mellitus, hypertension, chronic kidney disease, cirrhosis, immunosuppression, peptic ulcer disease, pregnancy, healthcare coverage, and epidemic year status.

To assess the cumulative burden of underlying conditions, a comorbidity burden variable was additionally constructed by summing the presence of diabetes mellitus, hypertension, peptic ulcer disease, chronic kidney disease, immunosuppression, and liver cirrhosis. Patients were categorized as having no comorbidities, one comorbidity, or two or more comorbidities. Pregnancy was not included in the comorbidity count.

Healthcare coverage was derived from the healthcare institution recorded in the national surveillance database. Institutions providing healthcare through the social security system (IMSS, ISSSTE, PEMEX, SEDENA, and SEMAR) were classified as social security; institutions providing public healthcare to populations without social security (SSA, IMSS-Bienestar, IMSS OPD, state and municipal health services, DIF, Red Cross, and university institutions) were classified as public uninsured system; and private healthcare institutions were classified as private. Dengue virus serotype was recorded in the surveillance database for cases in which serotyping was available and was classified as DENV-1, DENV-2, DENV-3, or DENV-4. Cases without an identified serotype were retained in the primary analyses and classified as having no serotype identified for descriptive purposes.

### 2.4. Definition of Epidemic Years

To evaluate the impact of periods of increased dengue transmission, a binary variable termed epidemic year was created. Years were classified according to the national dengue surveillance reports issued by the General Directorate of Epidemiology (Dirección General de Epidemiología, DGE) of the Mexican Ministry of Health. Dengue activity was assessed using the endemic channel methodology routinely employed by the DGE for national epidemiological surveillance. This method compares observed dengue transmission with expected historical patterns and classifies disease activity according to predefined transmission thresholds.

Years in which national dengue transmission consistently exceeded the upper expected limit of the endemic channel and generated nationwide epidemic activity were classified as epidemic years. Based on official surveillance reports and endemic channel analyses, 2023 and 2024 were classified as epidemic years, whereas 2020, 2021, 2022, and 2025 were classified as non-epidemic years [12,13].

To describe temporal patterns, the number of laboratory-confirmed dengue cases was calculated for each epidemiological week of symptom onset and plotted separately for each study year (2020–2025). This approach allowed direct visualization of annual differences in transmission magnitude and seasonal patterns without averaging across epidemic and non-epidemic years. Because the publicly available surveillance datasets had different annual cut-off dates, the final epidemiological weeks of some years may be incomplete. Weekly case counts were used exclusively for descriptive visualization of temporal transmission patterns and were not included as independent variables in the regression models.

### 2.5. Statistical Analysis

Descriptive statistics were used to summarize demographic, clinical, and epidemiological characteristics. Categorical variables were expressed as frequencies and percentages.

The geographic distribution of laboratory-confirmed dengue cases was described according to state of residence. A choropleth map was generated using Microsoft Excel (Microsoft 365, Microsoft Corporation, Redmond, WA, USA) to display the absolute number of laboratory-confirmed cases reported in each Mexican state during the study period.

Mortality proportions were calculated overall and stratified according to age group, hospitalization status, and epidemic-year status. Bivariate analyses were performed using Pearson’s chi-square test or Fisher’s exact test, as appropriate. Crude odds ratios (ORs) and their corresponding 95% confidence intervals (95% CIs) were estimated to evaluate associations between explanatory variables and study outcomes.

The distribution of identified dengue virus serotypes was examined by individual study year and according to epidemic-year status. Percentages were calculated among cases with an identified serotype. To assess whether changes in circulating serotypes influenced the association between epidemic years and hospitalization, a secondary analysis was conducted among cases with an identified viral serotype. A multivariable logistic regression model was fitted including epidemic-year status, DENV serotype, age group, healthcare coverage, diabetes mellitus, hypertension, peptic ulcer disease, chronic kidney disease, immunosuppression, liver cirrhosis, and pregnancy. DENV-1 was used as the reference serotype.

Multivariable binary logistic regression models were developed separately for hospitalization and mortality. The primary multivariable models included epidemic-year status, age group, healthcare coverage, diabetes mellitus, hypertension, peptic ulcer disease, chronic kidney disease, immunosuppression, liver cirrhosis, and pregnancy.

As a secondary analysis, a separate multivariable logistic regression model was fitted to evaluate the association between comorbidity burden and mortality. Comorbidity burden was entered as a categorical variable (none, one, or two or more comorbidities), with no comorbidities as the reference category. The model was adjusted for epidemic-year status, age group, healthcare coverage, and pregnancy. Individual comorbidities were not simultaneously included because they were used to construct the comorbidity burden variable.

Adjusted odds ratios (aORs) and 95% confidence intervals were estimated. Model performance was assessed using the Hosmer–Lemeshow goodness-of-fit test, Nagelkerke’s R^2^, and overall classification accuracy. A two-sided *p*-value < 0.05 was considered statistically significant.

All analyses were performed using IBM SPSS Statistics version 25.0 (IBM Corp., Armonk, NY, USA).

### 2.6. Ethical Considerations

This study was based exclusively on anonymized secondary data obtained from publicly available governmental databases. No personal identifiers were accessed and no direct contact with patients occurred. According to the Mexican Regulation of the General Health Law on Health Research, this study was classified as research without risk. The study protocol was reviewed and approved by the Local Research Committee and the Local Research Ethics Committee of the Mexican Institute of Social Security (IMSS) (approval No. R-2026-3605-023 Date 7 July 2026). The study was conducted in accordance with the ethical principles of the Declaration of Helsinki.

## 3. Results

A total of 235,226 laboratory-confirmed dengue cases reported in Mexico between 2020 and 2025 were included in the study. Females accounted for 55.3% of cases (*n* = 130,023). The most affected age group was 25–44 years (30.9%), followed by individuals aged 15–24 years (23.5%) and 5–14 years (23.4%). Most cases were reported through the public uninsured healthcare system (54.5%), while 43.5% were managed within social security institutions. Overall, 72.6% of cases occurred during epidemic years. A total of 90,661 patients (38.5%) required hospitalization, and 1604 deaths were recorded, corresponding to an overall mortality proportion of 0.68% (Table 1).

**Figure 1:**
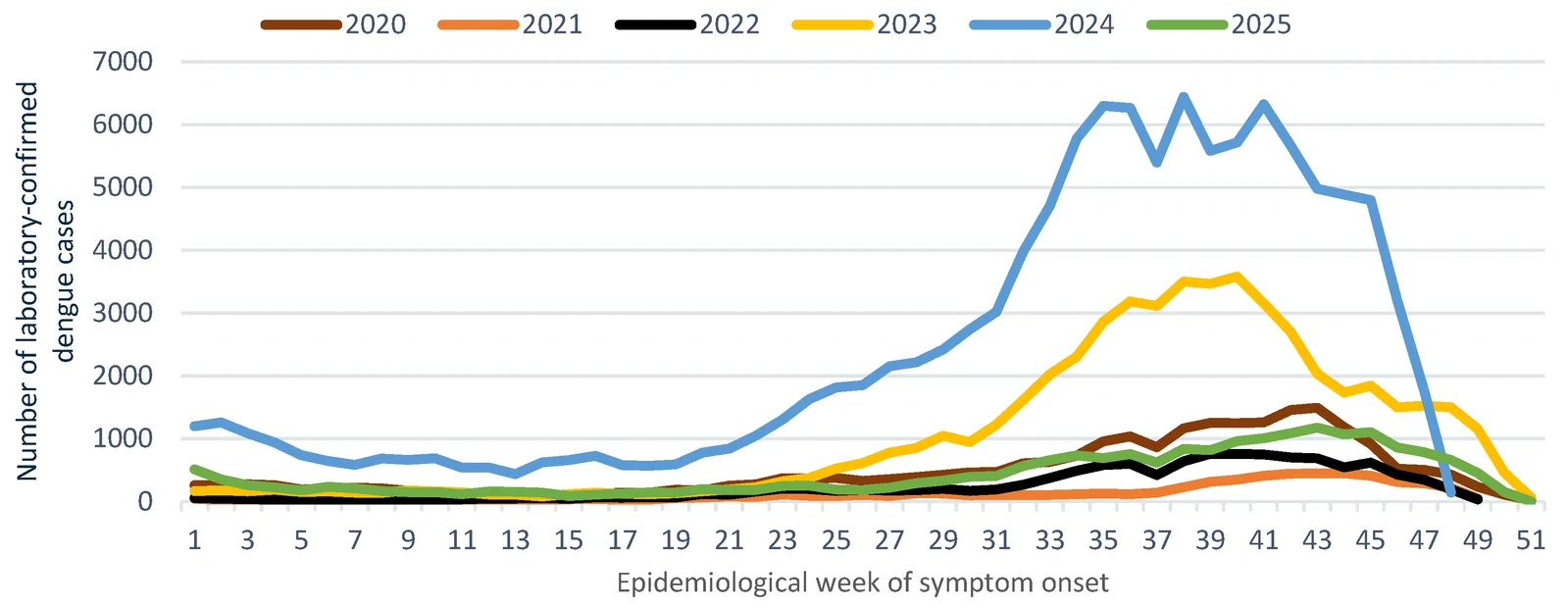
Weekly distribution of laboratory-confirmed dengue cases in Mexico, by epidemiological week and year, 2020–2025.

The weekly distribution of laboratory-confirmed dengue cases showed marked differences in transmission magnitude across study years (Figure 1). The largest epidemic activity was observed in 2024, with a pronounced increase beginning around epidemiological week 30 and the highest weekly case counts occurring between weeks 35 and 41. A similar seasonal pattern, although of lower magnitude, was observed in 2023. In contrast, weekly case counts were substantially lower during 2020–2022, while 2025 showed an intermediate pattern. Across years, the highest dengue activity was generally concentrated during the second half of the year.

Note: The curves represent reported laboratory-confirmed dengue cases by epidemiological week of symptom onset. The final weeks of some years are incomplete because publicly available surveillance databases had different annual cut-off dates.

Among the 103,186 cases with an identified serotype, marked temporal changes in serotype distribution were observed during the study period. DENV-2 predominated in 2020 (78.2%), 2021 (56.6%), and 2022 (57.0%), whereas DENV-3 became predominant in 2023 (59.0%) and accounted for 86.2% of identified serotypes in 2024. DENV-3 remained predominant in 2025 (91.8%) (Table S1). When grouped according to epidemic status, DENV-3 accounted for 78.3% of identified serotypes during epidemic years compared with 29.9% during non-epidemic years.

**Figure 2:**
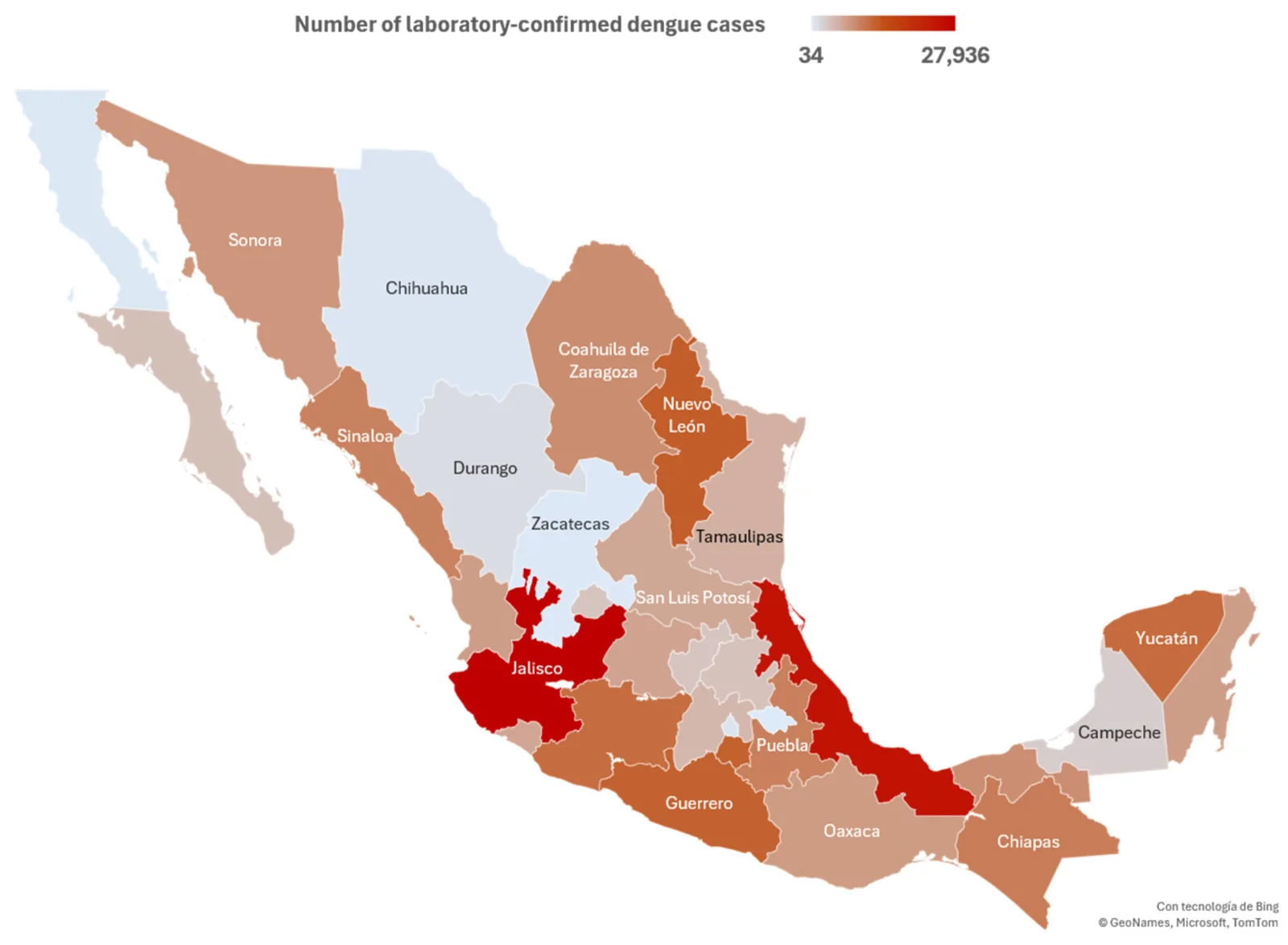
Geographic distribution of laboratory-confirmed dengue cases by state of residence, Mexico, 2020–2025.

Geographically, the largest numbers of laboratory-confirmed dengue cases were reported in Jalisco (27,936 cases; 11.9%), Veracruz (24,637; 10.5%), Nuevo León (12,712; 5.4%), Morelos (12,360; 5.3%), and Guerrero (12,240; 5.2%). Together, these five states accounted for approximately 38.2% of all laboratory-confirmed cases included in the analysis (Figure 2).

### 3.1. Clinical Outcomes According to Epidemic-Year Status

Of the 235,226 confirmed dengue cases, 170,819 (72.6%) occurred during epidemic years and 64,407 (27.4%) during non-epidemic years. Hospitalization was significantly more frequent during epidemic years than during non-epidemic years (40.2% vs. 34.1%; crude OR 1.30, 95% CI 1.27–1.32; *p* < 0.001). In contrast, mortality did not differ between epidemic and non-epidemic years (0.68% vs. 0.69%; crude OR 0.99, 95% CI 0.89–1.11; *p* = 0.919) (Table 2).

Among hospitalized patients, mortality was slightly lower during epidemic years than during non-epidemic years (1.64% vs. 1.88%; *p* = 0.016).

### 3.2. Factors Associated with Hospitalization

In bivariate analyses, epidemic-year status, age group, healthcare coverage, comorbidities, and pregnancy were associated with hospitalization (Table 3). In the multivariable model, epidemic-year status remained independently associated with hospitalization (aOR 1.26, 95% CI 1.24–1.29; *p* < 0.001). Chronic kidney disease (aOR 5.36, 95% CI 4.33–6.65), pregnancy (aOR 5.26, 95% CI 4.96–5.57), liver cirrhosis (aOR 3.19, 95% CI 2.38–4.28), and immunosuppression (aOR 2.79, 95% CI 2.21–3.53) showed the strongest associations with hospitalization. Healthcare coverage was also independently associated with hospitalization, with lower odds among patients receiving care through the public uninsured system (aOR 0.88, 95% CI 0.87–0.90) and higher odds among those receiving private care (aOR 2.49, 95% CI 2.34–2.65), compared with patients covered by social security.

In the secondary analysis restricted to cases with an identified viral serotype, the association between epidemic years and hospitalization persisted after additional adjustment for DENV serotype (aOR 1.19, 95% CI 1.15–1.23; *p* < 0.001). Compared with DENV-1, DENV-2 (aOR 1.94, 95% CI 1.84–2.04), DENV-3 (aOR 2.11, 95% CI 2.02–2.21), and DENV-4 (aOR 1.34, 95% CI 1.18–1.53) were independently associated with higher odds of hospitalization (all *p* < 0.001) (Table S2).

### 3.3. Factors Associated with Mortality

In contrast to hospitalization, epidemic-year status was not associated with mortality in either the bivariate (OR 0.99, 95% CI 0.89–1.11; *p* = 0.919) or multivariable analysis (aOR 1.00, 95% CI 0.90–1.12; *p* = 0.946) (Table 4). The strongest independent associations with mortality were observed for chronic kidney disease (aOR 6.98, 95% CI 5.23–9.32) and age ≥ 65 years (aOR 4.62, 95% CI 3.92–5.44), followed by liver cirrhosis (aOR 3.35, 95% CI 1.59–7.05), diabetes mellitus (aOR 2.69, 95% CI 2.25–3.21), and age < 5 years (aOR 2.26, 95% CI 1.75–2.91). Immunosuppression was associated with mortality in the bivariate analysis but was no longer statistically significant after multivariable adjustment (aOR 1.85, 95% CI 0.94–3.65; *p* = 0.076).

Mortality increased progressively with comorbidity burden, from 0.55% among patients without comorbidities to 2.98% among those with one comorbidity and 5.20% among those with two or more comorbidities. In a secondary multivariable analysis, compared with patients without comorbidities, those with one comorbidity had 3.65-fold higher adjusted odds of death (95% CI 3.12–4.28), whereas those with two or more comorbidities had 5.38-fold higher adjusted odds (95% CI 4.38–6.61; both *p* < 0.001). Epidemic-year status remained unassociated with mortality in this model (aOR 1.01, 95% CI 0.90–1.12; *p* = 0.922) (Table S3).

## 4. Discussion

In this nationwide study of 235,226 laboratory-confirmed dengue cases reported in Mexico between 2020 and 2025, epidemic-year status was independently associated with hospitalization but not with mortality. Patients diagnosed during epidemic years had 26% higher odds of hospitalization than those diagnosed during non-epidemic years after adjustment for age, healthcare coverage, comorbidities, and pregnancy. In contrast, epidemic-year status was not associated with an increased risk of death. To our knowledge, this is one of the first nationwide studies in Latin America to specifically evaluate whether epidemic transmission periods independently influence the risk of hospitalization and mortality among laboratory-confirmed dengue cases. These findings suggest that epidemic periods substantially increase healthcare demand and inpatient care utilization, whereas mortality was more strongly associated with patient-related characteristics than with epidemic-year status.

The Americas have experienced an unprecedented increase in dengue transmission during recent years, with record numbers of cases reported in 2023 and 2024 [1,3]. According to the World Health Organization and the Pan American Health Organization, the expansion of dengue has been driven by climate variability, urbanization, population growth, increased human mobility, and favorable ecological conditions for vector proliferation [1,3]. Recent analyses have highlighted the growing health and economic burden of dengue in Latin America, emphasizing the need to strengthen surveillance systems and healthcare preparedness during periods of increased transmission [2,3].

Changes in circulating DENV serotypes may also have contributed to the observed hospitalization patterns. A marked shift in serotype distribution occurred during the study period, with DENV-2 predominating among cases with an identified serotype during non-epidemic years and DENV-3 predominating during epidemic years. Previous evidence suggests that the relationship between DENV serotype and clinical severity is complex and may vary according to geographic setting and whether infection is primary or secondary [14]. In Mexico, a previous IMSS-based study conducted in 2023 found that DENV-3 was the predominant circulating serotype, whereas DENV-2, rather than DENV-3, was independently associated with severe dengue [15]. In our secondary analysis, DENV-3 was associated with higher odds of hospitalization compared with DENV-1. However, this finding should not be interpreted as evidence that DENV-3 intrinsically causes more severe disease, particularly because hospitalization and clinical severity represent distinct outcomes. Importantly, epidemic-year status remained independently associated with hospitalization after adjustment for viral serotype and other covariates (aOR 1.19, 95% CI 1.15–1.23), suggesting that changes in serotype distribution did not fully account for the increased hospitalization observed during epidemic years. Previous infection with a heterologous DENV serotype is also a recognized risk factor for severe dengue, potentially involving antibody-dependent enhancement and other immune mechanisms [16]. However, individual-level information on previous DENV infections and immune status was unavailable in our surveillance database; therefore, we could not determine whether secondary infections or underlying immunological mechanisms contributed to the observed hospitalization patterns.

In a retrospective cohort study conducted in Mexico, Fonseca-Portilla et al. reported that diabetes mellitus, chronic kidney disease, cirrhosis, and older age were independently associated with hospitalization and mortality among dengue patients [6]. Other studies have similarly highlighted the relevance of non-communicable diseases as determinants of dengue severity and mortality [7,8,9]. Our findings are consistent with these observations. Chronic kidney disease showed the strongest independent association with both hospitalization and mortality, while diabetes mellitus, liver cirrhosis, and other underlying conditions were also associated with increased odds of adverse outcomes. Beyond the associations with individual conditions, our analysis demonstrated a clear gradient according to comorbidity burden. Mortality increased from 0.55% among patients without comorbidities to 2.98% among those with one comorbidity and 5.20% among those with two or more comorbidities. After multivariable adjustment, patients with one comorbidity had 3.65-fold higher odds of death, whereas those with two or more comorbidities had 5.38-fold higher odds compared with patients without comorbidities. This graded association suggests that cumulative comorbidity burden may provide additional prognostic information beyond individual conditions and could help identify patients requiring closer clinical monitoring.

Age was also strongly associated with clinical outcomes, although the patterns differed between hospitalization and mortality. Compared with adults aged 25–44 years, children aged <5 years and those aged 5–14 years had the highest odds of hospitalization, whereas adults aged ≥65 years showed the strongest association with mortality. Children aged <5 years also had increased odds of death, indicating a higher vulnerability at both extremes of age. These findings are consistent with previous studies identifying young children, particularly infants, and older adults as populations at increased risk of adverse dengue outcomes [17,18]. The particularly strong association between advanced age and mortality may reflect reduced physiological reserve, a greater burden of underlying chronic conditions, and increased susceptibility to complications.

Unlike previous studies focused primarily on patient-level determinants, our investigation evaluated the influence of the epidemiological context by incorporating epidemic-year status as an explanatory variable. We found that hospitalization was significantly more frequent during epidemic years. Several mechanisms may explain this finding. Large outbreaks are commonly accompanied by increased healthcare-seeking behavior, intensified surveillance activities, and heightened clinical awareness among healthcare professionals. Furthermore, epidemic periods are characterized by a substantial increase in the number of symptomatic infections, generating greater demand for hospital services. Major dengue epidemics in Brazil and Sri Lanka have similarly demonstrated the substantial pressure that large outbreaks can place on healthcare systems and public health resources [19,20]. During epidemic periods, healthcare providers may also adopt a lower threshold for hospital admission because of uncertainty regarding disease progression and increased concern about severe outcomes. Consequently, hospitalization may reflect not only disease severity but also changes in clinical decision-making during periods of intense transmission.

Interestingly, epidemic-year status was not independently associated with mortality. Although hospitalization was more frequent during epidemic years, mortality rates remained stable after adjustment for demographic and clinical factors. This finding suggests that the increased burden imposed by epidemic periods does not necessarily translate into higher fatality among laboratory-confirmed cases. In our study, age and chronic comorbidities showed substantially stronger associations with mortality than epidemic-year status, consistent with previous evidence identifying individual susceptibility and underlying chronic conditions as major determinants of dengue mortality [6,7,8,9]. This interpretation is further supported by the slightly lower crude mortality observed among hospitalized patients during epidemic years compared with non-epidemic years.

Chronic kidney disease showed the strongest independent association with mortality, with nearly sevenfold higher odds of death and more than fivefold higher odds of hospitalization. This finding is consistent with previous studies from Mexico, Taiwan, and Brazil identifying chronic kidney disease as an important predictor of adverse dengue outcomes [6,21,22]. In a multicenter study from Taiwan, chronic kidney disease was independently associated with in-hospital mortality among adults with dengue [21], while a recent population-based analysis of more than 5.8 million dengue cases in Brazil found increased risks of hospitalization, severe dengue, and death among patients with chronic kidney disease [22]. Potential mechanisms underlying this association include chronic inflammation, endothelial dysfunction, impaired innate and adaptive immune responses, and reduced physiological reserve, which may increase vulnerability to organ dysfunction and hemodynamic instability during dengue infection.

Diabetes mellitus was independently associated with both hospitalization and mortality. This finding is consistent with the meta-analysis conducted by Htun et al., which demonstrated an increased risk of severe dengue among patients with diabetes [23], and with the study by Pang et al., who reported that diabetes and the coexistence of diabetes and hypertension were associated with dengue hemorrhagic fever during a predominantly DENV-2 epidemic [24]. More recently, Bisanzio et al. highlighted the growing public health relevance of the coexistence of dengue and diabetes, particularly in settings where both conditions are increasingly prevalent [25]. Potential mechanisms underlying these associations include chronic hyperglycemia, endothelial dysfunction, persistent low-grade inflammation, and alterations in innate and adaptive immune responses, which may increase vulnerability to adverse outcomes during dengue infection.

Pregnancy was strongly associated with hospitalization and was also independently associated with mortality after multivariable adjustment, although no significant crude association with mortality was observed. The higher likelihood of hospitalization may partly reflect closer clinical monitoring and differences in admission practices among pregnant women with dengue, rather than greater disease severity alone. Nevertheless, previous studies have identified pregnancy as a condition associated with an increased risk of severe dengue and adverse maternal outcomes. A surveillance-based study from Brazil reported a higher likelihood of severe dengue and death among pregnant women [26], while a systematic review and meta-analysis documented increased risks of maternal mortality and other adverse maternal and perinatal outcomes among women with dengue during pregnancy [27]. Therefore, the associations observed in our study should be interpreted as potentially reflecting both increased clinical vulnerability and differences in the management of dengue during pregnancy.

Healthcare coverage was independently associated with clinical outcomes. After adjustment for age, comorbidities, pregnancy, and epidemic-year status, patients in the public uninsured system and those receiving private care had higher odds of mortality than patients with social security coverage. In contrast, hospitalization patterns differed, with lower odds among patients in the public uninsured system and higher odds among those receiving private care compared with patients with social security coverage. These findings suggest that healthcare context may be associated with dengue outcomes beyond the demographic and clinical characteristics included in our models. However, healthcare coverage should not be interpreted as a direct measure of healthcare access or quality of care, and residual differences in access to services, referral patterns, healthcare-seeking behavior, case mix, and timing of presentation may have contributed to the observed associations.

This study has several strengths. It included a large number of laboratory-confirmed dengue cases identified through the national surveillance system and evaluated outcomes across epidemic and non-epidemic transmission periods. The nationwide scope of the study allowed assessment of the independent contribution of epidemic-year status to hospitalization and mortality across a large and geographically diverse population of laboratory-confirmed dengue cases. In addition, secondary analyses incorporating viral serotype and cumulative comorbidity burden provided further insight into the virological and clinical factors associated with adverse outcomes.

Nevertheless, several limitations should be acknowledged. Surveillance databases may be affected by underreporting and variability in healthcare-seeking behavior. The laboratory sampling strategy represents a potential source of selection bias because national surveillance procedures target 30% of dengue cases without warning signs for laboratory testing, compared with 100% of cases with warning signs or severe dengue. Consequently, laboratory-confirmed cases may overrepresent more severe clinical presentations and should not be considered representative of all notified dengue cases in Mexico. However, this sampling strategy remained unchanged throughout the study period, reducing the likelihood that changes in the prescribed sampling scheme itself explained the observed differences between epidemic and non-epidemic years. Nevertheless, variation in implementation, laboratory capacity, or surveillance intensity across years cannot be excluded.

Information regarding socioeconomic status, detailed clinical management, laboratory parameters, and individual-level measures of healthcare access was unavailable. Additionally, the surveillance database did not include standardized clinical classification of dengue severity according to World Health Organization criteria (dengue without warning signs, dengue with warning signs, and severe dengue), preventing direct assessment of the relationship between epidemic-year status and disease severity. Hospitalization was therefore evaluated as a distinct clinical outcome and should not be interpreted as equivalent to severe dengue, as admission decisions may also be influenced by age, pregnancy, comorbidities, healthcare setting, referral practices, and other clinical considerations. Likewise, information on warning signs, plasma leakage, laboratory abnormalities, and disease progression was unavailable, making it impossible to determine whether the increased hospitalization observed during epidemic years reflected greater disease severity or differences in clinical management and admission practices. Although death was recorded in the epidemiological surveillance database, the mortality outcome used in this study corresponded to deaths occurring among laboratory-confirmed dengue cases rather than to the final official classification of the cause of death. In Mexico, deaths associated with suspected or confirmed dengue cases undergo an official adjudication process to determine their attribution to dengue. Therefore, mortality findings should be interpreted as deaths recorded among laboratory-confirmed dengue cases and not necessarily as deaths directly attributable to dengue. Although the date of symptom onset was available, the database did not include the date of hospital admission or death, preventing assessment of the timing of hospitalization relative to disease onset, the interval from symptom onset to death, or potential differences in these intervals between epidemic and non-epidemic years. Consequently, we could not determine whether differences in the timing of clinical care or management contributed to the observed hospitalization and mortality patterns.

DENV serotype information was available for only 103,186 (43.9%) of the 235,226 laboratory-confirmed cases. Consequently, the serotype-adjusted analysis was restricted to this subset and may be subject to selection bias if cases with available serotype information differed systematically from those without serotype identification. Furthermore, information on previous DENV infections and individual immune status was unavailable, preventing direct assessment of secondary infections and immunological mechanisms such as antibody-dependent enhancement. In addition, genotype-level and genomic sequence information was unavailable, preventing assessment of changes in circulating viral lineages during the study period.

Healthcare coverage should also not be interpreted as a direct measure of healthcare access or quality, and residual confounding related to referral patterns, healthcare-seeking behavior, timing of presentation, and other unmeasured characteristics cannot be excluded.

The COVID-19 pandemic overlapped with the 2020–2022 study period and may have altered healthcare-seeking behavior, access to healthcare, surveillance practices, and hospitalization patterns. Previous evidence from Mexico has documented substantial changes in the reporting of notifiable diseases during the pandemic compared with pre-pandemic trends [28]. A comparable pre-pandemic baseline could not be included because individual-level open access surveillance datasets with the same structure and variables used in the present study were not available for years prior to 2020. Consequently, comparisons between the 2020–2022 period and subsequent years should be interpreted with caution. An additional limitation is that the publicly available surveillance databases had different annual data cut-off dates and did not contain complete records through 31 December for all years, particularly in December 2024. Consequently, the number of reported cases in the final days or weeks of some years may be underestimated, which should be considered when interpreting annual case counts and seasonal patterns. Finally, the observational nature of the study precludes establishing causal relationships.

In conclusion, epidemic years were independently associated with increased hospitalization among laboratory-confirmed dengue cases in Mexico but were not associated with increased mortality. Although substantial changes in circulating DENV serotypes occurred during the study period, epidemic-year status remained associated with hospitalization after adjustment for serotype, suggesting that the observed increase was not fully explained by changes in viral circulation. Mortality was strongly associated with age and underlying comorbidities, particularly chronic kidney disease, and increased progressively with greater comorbidity burden. These findings highlight the importance of strengthening hospital preparedness during epidemic periods while prioritizing close clinical monitoring of older adults, young children, pregnant women, and patients with chronic comorbidities who may be at increased risk of adverse outcomes.

## Figures and Tables

**Table 1:** Characteristics of laboratory-confirmed dengue cases included in the study, Mexico, 2020–2025.

Dimension	Characteristic	n	%
Sex	Female	130,023	55.3
	Male	105,203	44.7
Age group	<5 years	6432	2.7
	5–14 years	55,135	23.4
	15–24 years	55,211	23.5
	25–44 years	72,565	30.9
	45–64 years	35,987	15.3
	≥65 years	9896	4.2
Healthcare coverage	Social security	102,346	43.5
	Public uninsured system	128,254	54.5
	Private	4459	1.9
Epidemic status	Non-epidemic years	64,407	27.4
	Epidemic years	170,819	72.6
Patient management	Ambulatory	144,556	61.5
	Hospitalized	90,661	38.5
Vital status	Survived	233,622	99.3
	Died	1604	0.7
Comorbidities or condition	Diabetes mellitus	6567	2.8
	Hypertension	5329	2.3
	Peptic ulcer disease	113	<0.1
	Chronic kidney disease	597	0.3
	Immunosuppression	344	0.1
	Liver cirrhosis	217	0.1
	Pregnant	6121	2.6
DENV serotype	DENV-1	13,165	5.6
	DENV-2	22,220	9.4
	DENV-3	66,452	28.3
	DENV-4	1349	0.6
	No serotype identified	132,040	56.1

Note: Percentages were calculated using the total study population (N = 235,226). Hospitalization status was unavailable for 9 cases and healthcare coverage for 167 cases.

**Table 2:** Hospitalization and mortality according to epidemic-year status among laboratory-confirmed dengue cases, Mexico, 2020–2025.

Outcome	Non-Epidemic Years n (%)	Epidemic Years n (%)	Crude OR	Lower 95% CI	Upper 95% CI	*p*
Hospitalization	21,974 (34.1)	68,687 (40.2)	1.3	1.27	1.32	<0.001
Mortality	441 (0.69)	1163 (0.68)	0.99	0.89	1.11	0.919

**Table 3:** Factors associated with hospitalization among laboratory-confirmed dengue cases, Mexico, 2020–2025.

Variable	Crude OR (95% CI)	*p*-Value	Adjusted OR (95% CI)	*p*-Value
**Epidemic-year status**				
Non-epidemic year	Reference group	—	Reference group	—
Epidemic year	1.30 (1.27–1.32)	<0.001	1.26 (1.24–1.29)	<0.001
**Age group**				
25–44 years	Reference group	—	Reference group	—
<5 years	1.93 (1.83–2.03)	<0.001	2.21 (2.10–2.33)	<0.001
5–14 years	1.76 (1.72–1.80)	<0.001	1.99 (1.95–2.04)	<0.001
15–24 years	1.46 (1.43–1.50)	<0.001	1.50 (1.47–1.54)	<0.001
45–64 years	1.05 (1.03–1.08)	<0.001	1.02 (0.99–1.05)	0.24
≥65 years	1.89 (1.81–1.97)	<0.001	1.61 (1.54–1.69)	<0.001
**Healthcare coverage**				
Social security	Reference group	—	Reference group	—
Public uninsured system	0.93 (0.91–0.94)	<0.001	0.88 (0.87–0.90)	<0.001
Private	2.38 (2.24–2.53)	<0.001	2.49 (2.34–2.65)	<0.001
**Comorbidities**				
Diabetes mellitus	2.18 (2.07–2.29)	<0.001	2.15 (2.03–2.27)	<0.001
Hypertension	2.01 (1.90–2.12)	<0.001	1.62 (1.52–1.72)	<0.001
Peptic ulcer disease	2.80 (1.91–4.11)	<0.001	2.07 (1.38–3.11)	<0.001
Chronic kidney disease	7.25 (5.89–8.94)	<0.001	5.36 (4.33–6.65)	<0.001
Immunosuppression	3.14 (2.51–3.93)	<0.001	2.79 (2.21–3.53)	<0.001
Liver cirrhosis	3.22 (2.42–4.27)	<0.001	3.19 (2.38–4.28)	<0.001
Pregnancy	4.46 (4.22–4.72)	<0.001	5.26 (4.96–5.57)	<0.001

Note: OR, odds ratio; CI, confidence interval. Adjusted odds ratios were estimated using a multivariable logistic regression model including epidemic-year status, age group, healthcare coverage, diabetes mellitus, hypertension, peptic ulcer disease, chronic kidney disease, immunosuppression, liver cirrhosis, and pregnancy.

**Table 4:** Factors associated with mortality among laboratory-confirmed dengue cases, Mexico, 2020–2025.

Variable	Crude OR (95% CI)	*p*-Value	Adjusted OR (95% CI)	*p*-Value
Epidemic-year status				
Non-epidemic year	Reference group	—	Reference group	—
Epidemic year	0.99 (0.89–1.11)	0.919	1.00 (0.90–1.12)	0.946
Age group				
25–44 years	Reference group	—	Reference group	—
<5 years	2.21 (1.72–2.83)	<0.001	2.26 (1.75–2.91)	<0.001
5–14 years	0.99 (0.85–1.16)	0.912	1.04 (0.89–1.22)	0.606
15–24 years	0.68 (0.57–0.80)	<0.001	0.67 (0.57–0.80)	<0.001
45–64 years	1.75 (1.51–2.03)	<0.001	1.52 (1.31–1.78)	<0.001
≥65 years	6.68 (5.76–7.75)	<0.001	4.62 (3.92–5.44)	<0.001
Healthcare coverage				
Social security	Reference group	—	Reference group	—
Public uninsured system	1.27 (1.15–1.41)	<0.001	1.47 (1.32–1.63)	<0.001
Private	2.97 (2.33–3.78)	<0.001	2.34 (1.82–3.00)	<0.001
Comorbidities				
Diabetes mellitus	6.39 (5.56–7.34)	<0.001	2.69 (2.25–3.21)	<0.001
Hypertension	5.47 (4.66–6.41)	<0.001	1.35 (1.10–1.65)	0.004
Peptic ulcer disease	14.22 (7.42–27.27)	<0.001	3.04 (1.39–6.64)	0.005
Chronic kidney disease	21.90 (17.11–28.05)	<0.001	6.98 (5.23–9.32)	<0.001
Immunosuppression	5.76 (3.30–10.05)	<0.001	1.85 (0.94–3.65)	0.076
Liver cirrhosis	7.82 (4.26–14.38)	<0.001	3.35 (1.59–7.05)	0.001
Pregnancy	1.21 (0.91–1.60)	0.194	1.89 (1.41–2.54)	<0.001

Note: OR, odds ratio; CI, confidence interval. The multivariable model included epidemic-year status, age group, healthcare coverage, diabetes mellitus, hypertension, peptic ulcer disease, chronic kidney disease, immunosuppression, liver cirrhosis, and pregnancy. The reference categories were non-epidemic year, age 25–44 years, social security coverage, and absence of each comorbidity or pregnancy.

## Data Availability

The data analyzed in this study are publicly available administrative records. The source and description of the dataset are detailed within the Section 2 of this manuscript.

## Supplementary Materials

The following supporting information can be downloaded at: https://www.mdpi.com/article/10.3390/microorganisms14091920/s1. Table S1: Distribution of DENV serotypes among laboratory-confirmed dengue cases with available serotype information, Mexico, 2020–2025; Table S2: Multivariable logistic regression analysis of factors associated with hospitalization among laboratory-confirmed dengue cases with identified DENV serotype, Mexico, 2020–2025; Table S3: Association between cumulative comorbidity burden and mortality among laboratory-confirmed dengue cases, Mexico, 2020–2025.
