# Supplementary material for: Epidemic Years Increase Hospitalization but Not Mortality Among Laboratory-Confirmed Dengue Cases in Mexico: A Nationwide Surveillance Study, 2020–2025
Source: Microorganisms. 2026 Aug 31;14(9):1920. doi: 10.3390/microorganisms14091920 (PMC13608928; doi:10.3390/microorganisms14091920)
Supplement: Supplementary file 1 [file microorganisms-14-01920-s001.zip › microorganisms-4486004-supplementary.pdf]

Supplementary Materials

Epidemic years increase hospitalization but not mortality among laboratory-confirmed dengue cases in Mexico: a nationwide surveillance study, 2020–2025.

Table S1. Distribution of DENV serotypes among laboratory-confirmed dengue cases with available serotype information, Mexico, 2020–2025.

Year	DENV-1, n (%)	DENV-2, n (%)	DENV-3, n (%)	DENV-4, n (%)	Identified serotype, n
**2020**	2,524 (20.9%)	9,428 (78.2%)	100 (0.8%)	5 (<0.1%)	12,057
**2021**	1,367 (37.7%)	2,052 (56.6%)	194 (5.3%)	15 (0.4%)	3,628
**2022**	1,105 (17.8%)	3,541 (57.0%)	1,485 (23.9%)	77 (1.2%)	6,208
**2023**	3,616 (16.8%)	4,714 (22.0%)	12,666 (59.0%)	468 (2.2%)	21,464
**2024**	4,330 (8.3%)	2,181 (4.2%)	44,922 (86.2%)	680 (1.3%)	52,113
**2025**	223 (2.9%)	304 (3.9%)	7,085 (91.8%)	104 (1.3%)	7,716
**Total**	**13,165 (12.8%)**	**22,220 (21.5%)**	**66,452 (64.4%)**	**1,349 (1.3%)**	**103,186**

Note: Percentages were calculated among laboratory-confirmed dengue cases with an identified serotype within each year. Percentages in the Total row were calculated using all cases with an identified serotype (n=103,186) as the denominator. DENV, dengue virus.

Table S2. Multivariable logistic regression analysis of factors associated with hospitalization among laboratory-confirmed dengue cases with identified DENV serotype, Mexico, 2020–2025.

Variable	Adjusted OR (95% CI)	p-value
**Epidemic-year status**		
Non-epidemic year	Reference	—
Epidemic year	1.19 (1.15–1.23)	<0.001
**DENV serotype**		
DENV-1	Reference	—
DENV-2	1.94 (1.84–2.04)	<0.001
DENV-3	2.11 (2.02–2.21)	<0.001

Variable	Adjusted OR (95% CI)	p-value
DENV-4	1.34 (1.18–1.53)	<0.001
**Age group**		
25–44 years	Reference	—
<5 years	2.53 (2.33–2.75)	<0.001
5–14 years	2.26 (2.18–2.34)	<0.001
15–24 years	1.51 (1.45–1.56)	<0.001
45–64 years	0.99 (0.95–1.04)	0.724
≥65 years	1.74 (1.62–1.87)	<0.001
**Healthcare coverage**		
Social security	Reference	—
Public uninsured system	1.20 (1.16–1.23)	<0.001
Private	1.64 (1.48–1.81)	<0.001
**Comorbidities**		
Diabetes mellitus	2.19 (2.02–2.39)	<0.001
Hypertension	1.45 (1.32–1.59)	<0.001
Peptic ulcer disease	2.12 (1.17–3.81)	0.013
Chronic kidney disease	6.02 (4.34–8.35)	<0.001
Immunosuppression	3.73 (2.54–5.46)	<0.001
Liver cirrhosis	3.86 (2.46–6.05)	<0.001
Pregnancy	6.67 (6.13–7.26)	<0.001

Note: OR, odds ratio; CI, confidence interval; DENV, dengue virus. The analysis was restricted to laboratory-confirmed dengue cases with an identified DENV serotype. Adjusted odds ratios were estimated using a multivariable logistic regression model including epidemic-year status, DENV serotype, age group, healthcare coverage, diabetes mellitus, hypertension, peptic ulcer disease, chronic kidney disease, immunosuppression, liver cirrhosis, and pregnancy. Reference categories were non-epidemic year, DENV-1, age 25–44 years, social security coverage, and absence of each comorbidity or pregnancy.

Table S3. Association between cumulative comorbidity burden and mortality among laboratory-confirmed dengue cases, Mexico, 2020–2025.

Variable	Total, n	Deaths, n (%)	Adjusted OR (95% CI)	p-value
**Epidemic-year status**				
Non-epidemic year	64,407	441 (0.68%)	Reference	—
Epidemic year	170,819	1,163 (0.68%)	1.01 (0.90–1.12)	0.922
**Comorbidity burden**				
No comorbidities	224,807	1,237 (0.55%)	Reference	—
1 comorbidity	7,882	235 (2.98%)	3.65 (3.12–4.28)	<0.001
≥2 comorbidities	2,537	132 (5.20%)	5.38 (4.38–6.61)	<0.001
**Age group**				
25–44 years	72,565	381 (0.53%)	Reference	—
<5 years	6,432	74 (1.15%)	2.30 (1.78–2.96)	<0.001
5–14 years	55,135	287 (0.52%)	1.06 (0.91–1.24)	0.442
15–24 years	55,211	196 (0.36%)	0.68 (0.57–0.81)	<0.001
45–64 years	35,987	329 (0.91%)	1.40 (1.20–1.63)	<0.001
≥65 years	9,896	337 (3.41%)	4.12 (3.50–4.86)	<0.001
**Healthcare coverage**				
Social security	102,346	587 (0.57%)	Reference	—
Public uninsured system	128,254	934 (0.73%)	1.48 (1.33–1.65)	<0.001
Private	4,459	75 (1.68%)	2.30 (1.80–2.95)	<0.001
**Pregnancy**				
No	229,105	1,554 (0.68%)	Reference	—
Yes	6,121	50 (0.82%)	1.91 (1.42–2.55)	<0.001

Note: OR, odds ratio; CI, confidence interval. Mortality percentages were calculated within each category. Comorbidity burden was defined according to the number of recorded underlying conditions included in the study. Adjusted odds ratios were estimated using a multivariable logistic regression model including epidemic-

year status, comorbidity burden, age group, healthcare coverage, and pregnancy. Reference categories were non-epidemic year, no comorbidities, age 25–44 years, social security coverage, and absence of pregnancy.
